# The Effect of Different Postprandial Exercise Types on Glucose Response to Breakfast in Individuals with Type 2 Diabetes

**DOI:** 10.3390/nu13051440

**Published:** 2021-04-24

**Authors:** Alessio Bellini, Andrea Nicolò, Rocco Bulzomì, Ilenia Bazzucchi, Massimo Sacchetti

**Affiliations:** 1 Department of Movement, Human and Health Sciences, University of Rome “Foro Italico”, Piazza Lauro De Bosis 6, 00135 Rome, Italy; alessiobellini1@gmail.com (A.B.); andrea.nicolo@yahoo.com (A.N.); ilenia.bazzucchi@uniroma4.it (I.B.); 2 ASL RM 2, Centro di Diabetologia, Casa della Salute, Via Antistio 12, 00174 Rome, Italy; rocco.bulzomi@aslroma2.it

**Keywords:** combined exercise, aerobic exercise, resistance exercise, post-meal glycemia, circuit training, walking, glycemic peak

## Abstract

Postprandial exercise represents an important tool for improving the glycemic response to a meal. This study evaluates the effects of the combination and sequence of different exercise types on the postprandial glycemic response in patients with type 2 diabetes. In this repeated-measures crossover study, eight patients with type 2 diabetes performed five experimental conditions in a randomized order: (i) uninterrupted sitting (CON); (ii) 30 min of moderate intensity aerobic exercise (walking) (A); (iii) 30 min of combined aerobic and resistance exercise (AR); (iv) 30 min of combined resistance and aerobic exercise (RA); and (v) 15 min of resistance exercise (R). All the exercise sessions started 30 min after the beginning of a standardized breakfast. All the exercise conditions showed a significant attenuation of the post-meal glycemic excursion (*P* < 0.003) and the glucose incremental area under the curve at 0–120 min (*P* < 0.028) and 0–180 min (*P* < 0.048) compared with CON. A greater reduction in the glycemic peak was observed in A and AR compared to RA (*P* < 0.02). All the exercise types improved the post-meal glycemic response in patients with type 2 diabetes, with greater benefits when walking was performed alone or before resistance exercise.

## 1. Introduction

Postprandial hyperglycemia and high glucose excursions were identified as better predictors of cardiometabolic disorders than chronic fasting hyperglycemia in patients with type 2 diabetes [1,2]. Beyond pharmacological treatments, different lifestyle strategies can attenuate postprandial glycemic excursions. Among these, exercise is widely recognized as a valid tool for improving glycemic control and post-meal glycemic response, not only in patients with diabetes but also in healthy individuals [3,4,5,6]. Given that various exercise strategies can potentially be used to improve postprandial glycemia, it is important to understand how exercise should be prescribed for this purpose. While evidence suggests that exercise should be performed soon after the meal to reduce the hyperglycemic peak [7,8], less is known on how to select other exercise parameters, especially in patients with diabetes.

Among different exercise parameters considered for prescription in individuals with diabetes [9], the type of exercise has the potential to influence the postprandial glycemic response. Both aerobic and resistance exercises are effective in improving long-term glycemic control [3,10] and postprandial glycemia in individuals with type 2 diabetes [5,7,8]. However, the acute effect of resistance exercise on the postprandial glycemic response is much less studied than that of aerobic exercise, and only a few studies directly compared different exercise types [5,8].

The combination of aerobic and resistance exercises may even be more effective in terms of glycemic control than either aerobic or resistance exercise performed separately [11,12]. For instance, combined exercise elicits benefits on 24 h glycemic variability in patients with type 2 diabetes, both when aerobic exercise precedes resistance exercise and vice versa [13,14]. In individuals with type 1 diabetes, performing aerobic exercise before resistance exercise is seen to cause a greater acute glycemic decline than when using the opposite exercise sequence [15,16]. Although this notion is considered in order to reduce the risk of exercise-induced hypoglycemia, especially in patients with type 1 diabetes [15,17], it is also relevant when the desired effect is the acute attenuation of blood glucose levels. Therefore, it is conceivable that the order of aerobic and resistance exercises may play a role in modulating glycemia in the postprandial state, which is characterized by relatively high glucose and insulin levels. However, we are not aware of studies that investigated the effect of the order of combined aerobic and resistance exercises on the postprandial glycemic response in patients with type 2 diabetes, hence the need for further investigation.

Therefore, the purpose of this study was to evaluate the effects of the combination and sequence of aerobic and resistance exercises on the glycemic response to a standardized breakfast in individuals with type 2 diabetes, when performed in the early postprandial period. The exercise timing was set according to recommendations from previous studies [7,8]. Our findings were expected to shed some light on how to select the type of postprandial exercise in order to optimize the glycemic response to a meal in individuals with type 2 diabetes.

## 2. Materials and Methods

### 2.1. Participants

Nine sedentary (spending more than 8 h/day in sedentary behavior) and inactive (not reaching the minimum amount of exercise suggested by the guidelines [16,18]) individuals with type 2 diabetes voluntarily participated in this study. Among these individuals, eight participants (mean ± SD: age 62.6 ± 9.4 years; weight 87.6 ± 19.1 kg; height 1.7 ± 0.1 m; BMI 31.7 ± 5.2 kg/m^2^; HbA_1C_ 7.0 ± 0.3 %) completed all the experimental sessions. They were recruited from a diabetes clinic in Rome. Participants maintained the regular pharmacological treatment (6 metformin; 2 metformin + DPP4-I), as established with the physician. The study was conducted in accordance with the Helsinki Declaration and approved by the local Ethics Committee (17/2020). Informed consent was obtained from all volunteers involved in the study.

### 2.2. Study Design

**Figure 1:**
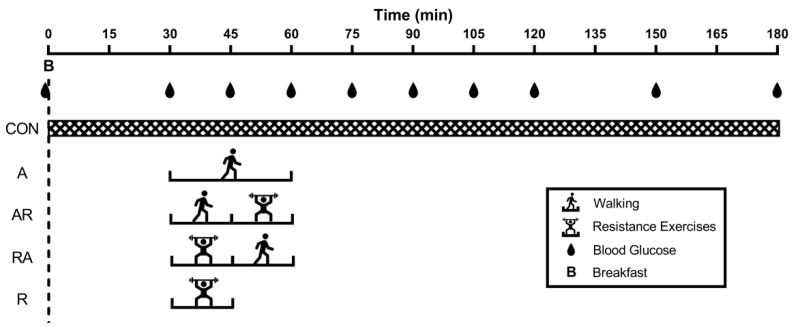
Graphic representation of the study design. Participants performed 30 min of walking (A), combined aerobic and resistance exercise (AR), combined resistance and aerobic exercise (RA), or 15 min of resistance exercise (R), starting 30 min after the beginning of the meal. A control (CON) condition was also performed.

The study was a randomized repeated-measures crossover study composed of a familiarization session and five experimental visits. Each visit lasted three hours and was separated by seven days. At the beginning of each visit, a standardized mixed breakfast was distributed to participants, after which they remained seated (CON) or performed aerobic exercise (A), resistance exercise (R), combined aerobic and resistance exercise (AR), or combined resistance and aerobic exercise (RA). Figure 1 shows a schematic representation of the study design.

### 2.3. Familiarization

During the preliminary visit, participants were familiarized with all the exercise types and the experimental procedures. Furthermore, some recommendations were made for the kinds of nutritional and exercise behavior to be adopted before each visit. Specifically, participants were invited to avoid alcohol consumption in the evening before, as well as caffeine and smoking in the morning of each session. In addition, they were asked to refrain from performing moderate-to-vigorous intensity exercise during the 48 h preceding each visit. They were also asked to register the activities they performed and the diet they followed on the day before the first session and to replicate these before the remaining visits.

### 2.4. Breakfast

After fasting overnight (≥10 h), participants arrived at the laboratory at 07.30 a.m., and a standardized breakfast was provided at 08.00 a.m. The breakfast consisted of rusks, partially skimmed milk, and fruit jam, which provided 310 kcal (66% carbohydrate, 19% fat, 15% protein). During the visits, participants were allowed to drink water ad libitum, but no other food or drinks were consumed.

### 2.5. Exercise and Sitting Time

The participants performed five experimental visits in a randomized order. After breakfast, participants remained seated during the whole experimental period (CON) or performed 15 or 30 min of exercise, starting 30 min after the beginning of the meal. More precisely, the participants performed the following: (i) 30 min of aerobic exercise (A); (ii) 15 min of aerobic exercise followed by 15 min of resistance exercise (AR); (iii) 15 min of resistance exercise followed by 15 min of aerobic exercise (RA); or (iv) 15 min of resistance exercise (R).

The aerobic exercise consisted of brisk walking at 100 steps per minutes. A digital metronome (Soundbrenner, Berlin, Germany) was used to establish step cadence, which is used as measure of walking intensity. Step cadence can be easily reproduced, and it is recognized as a valid estimation of the metabolic cost of exercise [19].

The resistance exercise consisted of circuits of five exercises: squatting with a medicine ball, rowing with an elastic band, stepping up, military presses with an elastic band, and a standing isometric plank. Each exercise lasted 30 s and was followed by 30 s of passive rest. The sequence of the 5 different exercises was repeated three times for a total duration of 15 min.

The participants were asked to remain seated and to limit movements during CON and after the exercise period. They were allowed to read, use the computer, or perform other cognitive activities while sitting, and they were requested to replicate the same activities during all the visits.

### 2.6. Glycemic Assessment

Glycemia was monitored using capillary blood samples collected through the fingertips method. Glucose analysis was performed using glucose monitors and reactive strips (Contour^®^ Next, Bayer HealthCare S.p.A., Milan, Italy), previously validated by Klaff et al. [20]. Blood glucose was measured every 15 min during the first 2 h and every 30 min during the last hour (Figure 1). Two samples were collected for the baseline measurement (at 0 min), and the average of the two measurements was considered (coefficient of variation < 2%). When a difference of >10% was found between the two values, a third sample was analyzed. Procedures were strictly controlled to prevent any alteration in the glucose measurements related to external factors.

### 2.7. Calculation and Statistical Analysis

All statistical analyses were performed using IBM SPSS software version 23.0 (SPSS Inc, Chicago, IL, USA). The data were checked for normality before statistical analysis. A two-way repeated measures ANOVA (condition × time) was carried out to compare the time course of glycemia between conditions. In the case of significant differences, a one-way repeated measures ANOVA was performed to determine differences between conditions at each time point. The time-averaged positive incremental area under the curve (iAUC) was calculated at three time frames (0–60 min, 0–120 min, and 0–180 min) [21]. A one-way repeated measures ANOVA was used to evaluate differences between conditions for the iAUC. In addition, a 0–180 mean glucose concentration was also calculated and analyzed using a one-way repeated measures ANOVA. The degrees of freedom of the within-subject comparisons were adjusted using the Greenhouse–Geisser correction for ε < 0.75, and the Huynh–Feldt correction for ε > 0.75. When significant differences were found, multiple comparisons were analyzed using the least significant differences (LSD) correction. The statistical significance level was set at 0.05. Partial eta squared (*η*_p_^2^) effect sizes were determined, considering *η*_p_^2^ ≥ 0.01 as small, *η*_p_^2^ ≥ 0.059 as medium, and *η*_p_^2^ ≥ 0.138 as large [22]. All values are reported as mean (±SEM) in Figures and as mean (±SD) in the text.

## 3. Results

**Figure 2:**
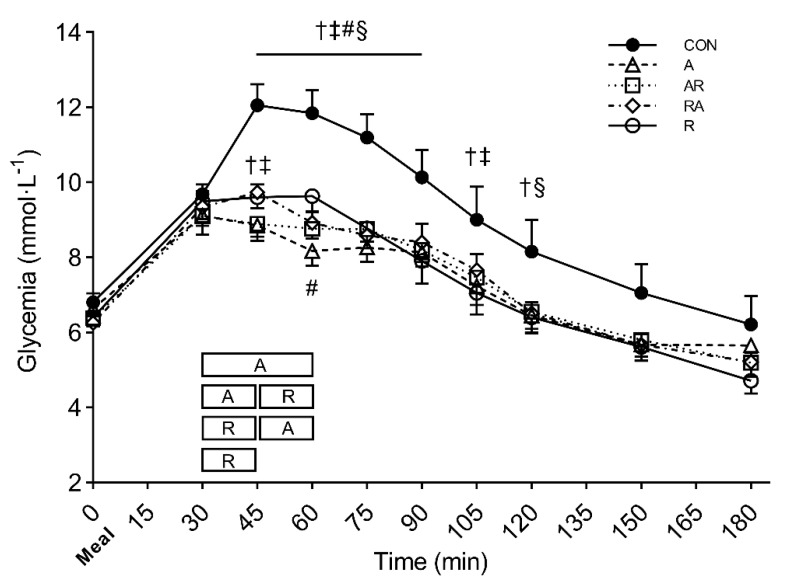
Glycemic time course of the five experimental conditions. Symbols: †, *P* < 0.05 vs. A; ‡, *P* < 0.05 vs. AR; #, *P* < 0.05 vs. RA; §, *P* < 0.05 vs. R. The boxes in the graphs illustrate the exercise bout and the arrows illustrate the meal. Values are reported as mean (±SEM).

The comparison of the time course of glucose concentration across conditions showed a significant interaction (condition × time) (*P* = 0.005, *η*_p_^2^ = 0.370). A similar time course was found for all the exercise conditions, showing a lower glucose concentration in the postprandial period compared with CON. The postprandial glycemic peak observed in the control condition (at 45 min) was significantly reduced in all the exercise conditions (*P* < 0.003). In addition, at the same time point (i.e., 45 min), A and AR showed significantly lower glucose values than RA (*P* = 0.019 and *P* = 0.006, respectively). Further details on the simple main effect of condition at each time point are shown in Figure 2.

**Figure 3:**
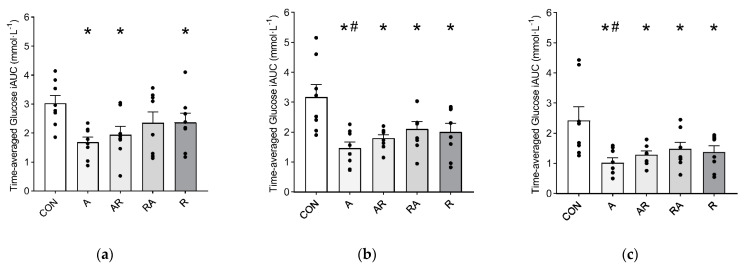
Time-averaged positive iAUC at: (**a**) 0–60 min; (**b**) 0–120 min; (**c**) 0–180 min. Symbols: * *P* < 0.05 vs. CON; # *P* < 0.05 vs. RA. Values are reported as mean (± SEM).

Glucose iAUC was significantly attenuated at 0–60 min in all the exercise conditions as compared with CON (*P* < 0.045), except for RA. Significantly lower values were observed at 0–120 min (*P* < 0.028) and 0–180 min (*P* < 0.048) in all the exercise conditions as compared with CON. Likewise, A showed a significantly lower glucose iAUC compared with RA at 0–120 min and 0–180 min (*P* = 0.036 and *P* = 0.038, respectively) (Figure 3).

The mean glucose concentration at 0–180 min showed significant between-condition differences (*P* = 0.004, *η*_p_^2^ = 0.515). Post hoc multiple comparisons revealed significantly lower values in all the exercise conditions (A: 7.42 ± 0.76 mmol·L^−1^; AR: 7.51 ± 0.63 mmol·L^−1^; RA: 7.63 ± 0.53 mmol·L^−1^; R: 7.56 ± 0.89 mmol·L^−1^;) as compared with CON (9.21 ± 1.60 mmol·L^−1^) (*P* < 0.016).

## 4. Discussion

Postprandial glycemic excursions are major predictors of cardiometabolic disorders in type 2 diabetes individuals [1,2], and exercise is an important tool for attenuating these excursions [5,7,8]. In the present study, we evaluated the effect of different postprandial exercise types on glycemic response after a standardized breakfast in individuals with type 2 diabetes. We compared the effect of combined aerobic and resistance exercises performed in different sequences with that of aerobic and resistance exercises performed in separate sessions. We found that all the exercise conditions significantly improved the postprandial glycemic response as compared with prolonged sitting. In addition, we observed a greater reduction in the postprandial glycemic response when aerobic exercise was performed alone or when it preceded the resistance exercise. These findings have implications for the prescription of postprandial exercise to patients with type 2 diabetes.

When prescribing postprandial exercise, it is important to choose the correct timing after the meal, especially in patients with type 2 diabetes that usually reach relatively high values of glucose peak. In this view, to maximize its effect, exercise should start soon after the beginning of the meal and before the glucose peak occurs [7,8,23,24]. This is why we selected an exercise timing of 30 min for our diabetic volunteers. Our findings show that this strategy was effective as we found a significant and substantial attenuation of the post-meal glycemic excursion in all the exercise conditions tested. Hence, our results further confirm the crucial role of exercise timing in attenuating the postprandial glycemic response, with a possible reduction in the negative consequences associated with hyperglycemic excursions.

All the exercise modalities tested significantly reduced the postprandial glycemic peak and the glucose iAUC response at 0–120 min and 0–180 min as compared with the control condition. While the benefits of postprandial aerobic exercise on post-meal glycemia were extensively reported in previous literature, fewer studies were conducted on resistance exercise [5,7,24]. Given that resistance exercise may generally require higher levels of effort compared to aerobic exercise, we tested the effects of a relatively short bout of circuit resistance training, which can be regarded as a feasible strategy to improve exercise adherence in patients with type 2 diabetes. We observed that 15 min of resistance exercise (circuit training), targeting the muscles of the lower and upper body significantly attenuated the postprandial glucose response. Benefits of resistance exercise were already observed in the first hour after the meal, where a significant reduction in the iAUC was found as compared to the control condition. The glycemic response in the first hour post-load was previously identified as an important predictor of cardiometabolic disorders [25], and the reduction observed with resistance exercise may have implications for the prevention of these disorders in patients with diabetes. Our results are in accordance with previous investigations showing the beneficial effects of postprandial resistance exercise on the metabolic response to a meal in type 2 diabetes patients [26,27,28]. However, it is interesting to note that we observed improvements in the post-meal glucose response with only 15 min of circuit exercise training, while the positive effect of exercise on postprandial glycemia was observed with 45 min [27], or with 60 min [26] of traditional resistance exercise in previous studies. Hence, our results suggest that 15 min of circuit resistance training started soon after a meal is a feasible and time-effective strategy for a rapid and sustained attenuation of blood glucose level during the postprandial period in these patients. The importance of resistance exercise as a time-effective strategy is also confirmed by the similar effect in the post-meal glycemic response found when comparing 15 min of resistance exercise with the same exercise followed by 15 min of aerobic exercise (RA). Indeed, neither the analysis of the glycemic peak nor the glucose iAUC response showed significant differences between these two exercise types. This suggests that the additional 15 min of moderate-intensity walking during the combined session did not further improve the postprandial glycemic response as compared to 15 min of resistance circuit training alone.

Despite the improvement of the glycemic response observed in resistance exercise, our findings suggest that additional benefits can be obtained if the exercise bout starts with aerobic exercise. Indeed, a greater attenuation of the glycemic peak was observed when aerobic exercise was performed before resistance exercise (AR), rather than vice versa (RA). Furthermore, significantly lower glucose iAUC values were observed at two and three hours after the meal for aerobic exercise performed alone as compared with combined resistance and aerobic exercise (RA). Our results highlight the hypoglycemic effect of aerobic exercise when performed alone or before a resistance exercise bout, as already suggested in type 1 diabetes individuals [15,16]. A different hormonal response may be responsible for the different effect on glycemia when the order of aerobic and resistance exercise is changed. More specifically, an enhanced secretion of the growth hormone was observed in previous studies when the resistance bout preceded the aerobic one, thus counteracting the hypoglycemic effect of the exercise session [29]. However, the mechanisms underlying the effects of different sequences of combined exercises on the postprandial glycemic response may differ between patients with type 1 and type 2 diabetes, and they may be influenced by the pathological and nutritional status of patients. Hence, further studies are needed for evaluating the mechanisms related to the effects of exercise on post-meal glycemia in patients with type 2 diabetes. Our findings highlight the efficacy of different exercise types in improving post-meal glycemic control, although aerobic exercise seems to offer greater benefits.

The present study did not focus on the mechanisms underlying the hypoglycemic effect of exercise. However, it was previously found that exercise stimulates insulin-dependent and insulin-independent glucose uptake [30,31] and may influence gastric emptying, the rate of glucose appearance, and endogenous glucose production [32,33]. In addition, aerobic, resistance, and combined exercises may elicit, to some extent, a different hormonal response [29], thus potentially explaining some of the between-condition differences observed in glycemia in the present study. For instance, it is conceivable that these exercise types may have different effects on the growth hormone and insulinemic response. Future studies are required to better clarify the mechanisms underlying the benefits of different postprandial exercise types found in the post-meal glycemic response of patients with type 2 diabetes.

In conclusion, the glycemic response to a standardized breakfast in individuals with type 2 diabetes was improved by either aerobic exercise, resistance exercise, or combined exercise started 30 min after the meal. Performing aerobic exercise alone or before resistance exercise provided the largest attenuation of the post-meal glycemic peak. Nonetheless, 15 min of circuit resistance training exercise provided a sufficient stimulus for attenuating the post-meal glycemic response. These results have implications for postprandial exercise prescription and adherence in these patients. Considering the cost–benefit ratio of the different types of exercise tested, it appears that brisk walking is probably still the simplest but effective option for the management of postprandial glycemia in individuals with type 2 diabetes, especially in older and/or deconditioned individuals. On the other hand, our results clearly indicate that even resistance exercise, also performed in circuits, as well as combined exercise, can still be counted among the tools available for postprandial metabolic control. This increases the options available to patients and exercise professionals as they may choose from among several exercise types according to their abilities, preferences, and also the complications derived from the disease. Future studies should investigate the mechanisms underlying the effects of different exercise types on postprandial glycemia and whether these effects may vary when performing exercise at different timings and times of the day.

## Data Availability

The data presented in this study are available on request from the corresponding author.
